# Diminished health and social outcomes among men who have sex with men who use drugs in Zimbabwe

**DOI:** 10.4102/sajhivmed.v24i1.1513

**Published:** 2023-09-27

**Authors:** Munyaradzi Mapingure, Innocent Chingombe, Tafadzwa Dzinamarira, Chesterfield Samba, Brian Moyo, Owen Mugurungi, Godfrey Musuka

**Affiliations:** 1 Department of Strategic Information, ICAP at Columbia University, Harare, Zimbabwe; 2 Gays and Lesbians Association of ZImbabwe (GALZ), Harare, Zimbabwe; 3 AIDS and TB Programmes, Ministry of Health and Child Care, Harare, Zimbabwe; 4 International Initiative for Impact Evaluation, Harare, Zimbabwe

Drug and substance use, which is the use of injectable and non-injectable drugs or medicines for pleasure or reasons not recommended by a doctor, is a growing epidemic across the globe, including Zimbabwe. Evidence suggests an upward trend of substance use in the Zimbabwean population.^1^ Media reports of youths identified to be in a drunken stupour are widespread, painting an escalating and worrying situation of substance use in the country.^2,3^ This can be attributed to increased poverty and dwindling economic opportunities that have left many young people idle and depressed, only to find solace in illicit substance use.^4^ Drug and substance use in Zimbabwe is now rated 8th in the country’s top 10 risk factors accounting for disability-adjusted life years lost (DALYs).^5^

The global HIV epidemic among men who have sex with men (MSM) continues to be a significant public health concern worldwide, given the disproportionate burden of infection this group faces.^6^ HIV prevalence in Zimbabwe remains one of the highest globally at 11.6% among the general population^7^ and is even higher at 23.4% among MSM.^8^

Evidence points to high-risk sexual health behaviours and poor health outcomes among drug users. Compared to other persons, HIV-positive people who use drugs were found to experience poorer clinical outcomes, such as antiretroviral therapy (ART) initiation and sustained viral suppression, and higher mortality rates.^9^

Data from our study on HIV and sexually transmissible infection (STI) biobehavioural surveys (BBS) among MSM in Zimbabwe^7^ were used to explore the sexual behaviours and health outcomes among MSM who use drugs in Zimbabwe. Drug use definition was use of injectable or non-injectable drugs or medicine for pleasure or for reasons not recommended by a doctor. We hypothesised that MSM who use drugs have poor health and social outcomes compared to those who do not use drugs. The study was a cross-sectional BBS using respondent-driven sampling. Consenting participants completed a questionnaire on sociodemographic and HIV risk behaviours. They underwent biomarker testing, including rapid testing for HIV, hepatitis B (HBV), and syphilis. HIV-positive patients were tested for CD4, viral load, and recent HIV infection via the rapid test for recent HIV infection. STATA^®^ statistical package version 17 (StataCorp LLC, College Station, Texas, United States [US]; 2021) was used to compare differences between people who use drugs and those who do not. Chi-square tests were used for categorical variables, while Student’s *t*-test was used for continuous variables. The significance level was set at *P* = 0.05. Simple proportions were used for descriptive statistics.

The prevalence of drug use was 668/1548 (43%); see Table 1. Unemployment prevalence was higher among those who use drugs (40% vs 36%, *P* = 0.001), and their earnings were significantly lower ($129.00 vs $209.00, *P* = 0.017). While only small fractions of either cohort reported being paid with money or goods the first time they had sex with a man, this percentage was higher among those who use drugs compared to those who did not (6.3% vs 2.1%, *P* = 0.001). The same pattern is shown for exchanging money or goods for sex in their lifetimes. Those who use drugs were more likely to report that they do not use condoms during sex when drunk (35.6% vs 21.8%, *P* = 0.001). More of those who use drugs have been selling sex for longer (6.7 vs 4.8 years, *P* = 0.032), and they have a higher percentage misconception that a healthy-looking person cannot have HIV (7.2% vs 4.3%, *P* = 0.013). Fewer men who use drugs were ever tested for HIV (81.3% vs 88.7%, *P* = 0.001). Although not statistically significant, a smaller percentage of drug users disclosed their HIV status to anyone. Overall > 90% went for care after testing positive for HIV, and the percentages were similar between the two groups (91.1% vs 94.3%, *P* = 0.441). A smaller percentage of those who have ever used drugs have ever taken pre-exposure prophylaxis (PrEP) (26.0% vs 32.4, *P* = 0.099). HIV prevalence was high among MSM who do not use drugs (25.5%) compared to those who do (18.8%). The recency of HIV infection was equally high in MSM not using drugs compared to those using drugs (13.4% vs 5.7%, *P* = 0.027). CD4 count and percentage of viral load suppression was not statistically different between HIV-positive MSM who use drugs (433 vs 451, *P* = 0.464) and those who do not (59.4% vs 62.7%, *P* = 0.545).

**TABLE 1:** Sexual health behaviours and health outcomes among MSM who use drugs in Zimbabwe.

Variable	MSM who use drugs (*n* = 668)	MSM who do not use drugs (*n* = 870)	*P*
Age in years	28.2	27.8	0.026
Average monthly income in USD	127.00	209.00	0.017
Percentage unemployed	40.0	36.0	0.001
Age in years of sexual debut with women	17.6	18.8	0.001
Number of lifetime female sexual partners for MSM	10	8	0.020
Age in years of sexual debut with men	20.4	20.6	0.631
Number of lifetime male sexual partners for MSM	22	13	0.083
Percentage who paid money or goods the first time they had sex with a man	6.3	2.1	0.001
Percentage who received money or goods the first time they had sex with a man	11.8	8.0	0.013
Percentage who paid money or goods to have sex in their lifetime	19.5	10.7	0.001
Percentage who ever received money or goods to have sex	21.6	15.9	0.005
The average number of male partners in the last six months	3.4	2.9	0.118
Percentage of condom use at last sex	66.9	67.2	0.912
Percentage who report non-use of condoms when drunk	35.6	21.8	0.001
Percentage who report non-use of condoms when offered more money	7.6	5.8	0.208
Percentage who used a condom while having an STI symptom	44.8	52.8	0.500
Number of years he has been exchanging money, goods or services for sex	6.7	4.8	0.032
Percentage with the misconception that a healthy-looking person cannot have HIV	7.2	4.3	0.013
Prevalence of depression	41.9	46.9	0.052
Percentage ever tested for HIV	81.3	88.7	0.001
Percentage who disclosed their HIV status	1.8	5.7	0.244
Percentage who went for HIV care after testing HIV-positive	91.1	94.3	0.441
Percentage who have ever taken PrEP	26.0	32.4	0.099
Percentage with TB symptoms in the last 12 months	17.7	16.2	0.817
Percentage who tested sputum positive among those who had TB symptoms	60.0	20.0	0.197
Percentage who had an ulcer or sore on penis in the last 12 months	4.4	2.2	0.016
Percentage who had pain during urination	12.3	9.2	0.05
Percentage who visited a health facility for treatment after having an STI	56.4	64.2	0.172
HIV prevalence (%)	18.8	25.5	0.002
Percentage of recency of infection; that is, infection acquired in the last 12 months	5.7	13.4	0.027
Mean CD4 count (cells/µL of blood) among those who tested HIV-positive in the study	433	451	0.464
Prevalence of syphilis (%)	5.2	6.2	0.420
Percentage of viral load suppression (< 1000 RNA copies per mL of blood among those HIV-positive)	59.4	62.7	0.545

MSM, men who have sex with men; USD, United States dollars; STI, sexually transmissible infection; PrEP, pre-exposure prophylaxis.

People who use drugs are more likely to experience social determinants of poor health, including unemployment or being engaged in lower-paying jobs, which may be interrelated and also, in turn, could exacerbate their substance use patterns.^10^

Antiretroviral therapy in people living with HIV improves clinical outcomes and reduces onward transmission risk through viral suppression.^11^ HIV non-suppression in people living with HIV who use drugs is mainly attributable to sub-optimal ART adherence, which increases HIV incidence.^12^ Pre-exposure prophylaxis, which has been shown to reduce HIV transmission, was underutilised in this study sample.^13^

Other adverse health consequences of drug and alcohol use are poorer self-rated health, more physical symptoms, lower life satisfaction and poorer mental health and social functioning.^14,15^

In this study, we found diminished health and social outcomes among HIV-positive MSM who use drugs in Zimbabwe. Drug use undoubtedly shapes sexual health risk, as evidenced by higher risk behaviours shown in this study sample among drug users. Drug use is associated with an increased likelihood of engaging in behaviours that elevate the risk of HIV acquisition, which has been reported elsewhere.^16^ People who use drugs are disproportionately affected by comorbidities, such as hepatitis C, skin abscesses, and endocarditis, which can lead to adverse HIV clinical outcomes.^17^ Several other studies have found that infection with HIV and other STIs is more likely among MSM who use recreational drugs than those who do not.^9,18^ We, however, did not find this link in our study.

In conclusion, the world is now faced with a growing epidemic of drug use. People who use drugs engage in risky sexual health behaviours and experience poor health outcomes. This knowledge should be applied toward developing multilevel interventions such as increasing anti-drug use campaigns and strategies to keep men from using. There is a need to establish more detoxification centres to ease drug dependence and treat the disorder. There is an urgent need to address the burden of negative drug use consequences, and this is in synergy with the goals of the World Health Organizaton Special Initiative for Mental Health.^19^

## References

[1] Mukwenha S, Murewanhema G, Madziva R, Dzinamarira T, Herrera H, Musuka G. Increased illicit substance use among Zimbabwean adolescents and youths during the COVID-19 ERA: An impending public health disaster. Addiction. 2021;117(4):1177–1178. 10.1111/add.15729 34729833

[2] Nhunzvi C. Substance abuse: Beyond peer pressure, and experimentation [homepage on the Internet]. The Herald. 24 October 2019. [cited 2022 Jul 06]. Available from: https://www.herald.co.zw/substance-abuse-beyond-peer-pressure-experimentation

[3] Chingono N. ‘It’ll kill me’: Zimbabwe counts cost of rise in illicit alcohol use [home page on the Internet]. The Guardian. 27 September 2021 [cited 2022 May 12]. Available from: https://www.theguardian.com/global-development/2021/sep/27/itll-kill-me-zimbabwe-counts-cost-of-rise-in-illicit-alcohol-use

[4] Factsheet–Zimbabwe’s drug, alcohol use problem under COVID-19 [homepage on the Internet]. ZIMFACT. 10 August 2021 [cited 2023 May 18]. Available from: https://zimfact.org/factsheet-zimbabwes-drug-alcohol-use-problem-under-covid-19/

[5] Institute for Health Metrics and Evaluation (IHME). Zimbabwe profile [homepage on the Internet]. Seattle, WA: IHME, University of Washington; 2021 [cited 2023 Jun 01]. Available from: http://www.healthdata.org/Zimbabwe

[6] Mgbako O, Park SH, Callander D, et al. Transactional sex, condomless anal sex, and HIV risk among men who have sex with men. Int J STD AIDS. 2019;30(8):795–801. 10.1177/0956462418823411 31142221 PMC7199436

[7] Ministry of Health and Child Care (MoHCC). Zimbabwe Population-based HIV Impact Assessment (ZIMPHIA 2020): Final Report. Harare; 2021.

[8] ICAP at Columbia University. HIV and STI biobehavioral survey among men who have sex with men, transgender women, and genderqueer individuals in Zimbabwe – Final Report. ICAP at Columbia University: New York, NY; 2020.

[9] Dasgupta S, Tie Y, Lemons-Lyn A, Broz D, Buchacz K, Shouse RL. HIV-positive persons who inject drugs experience poor health outcomes and unmet needs for care services. AIDS Care. 2021;33(9):1146–1154. 10.1080/09540121.2020.1826396 32985227 PMC8628508

[10] Nandi A, Glass TA, Cole SR, et al. Neighborhood poverty and injection cessation in a sample of injection drug users. Am J Epidemiol. 2010;171(4):391–398. 10.1093/aje/kwp416 20093307 PMC2877451

[11] Levintow SN, Pence BW, Powers KA, et al. Depression, antiretroviral therapy initiation, and HIV viral suppression among people who inject drugs in Vietnam. J Affect Disord. 2021;281:208–215. 10.1016/j.jad.2020.12.024 33333474 PMC7855445

[12] Cohen MS, Chen YQ, McCauley M, et al. Prevention of HIV-1 infection with early antiretroviral therapy. N Engl J Med. 2011;365(6):493–505. 10.1056/NEJMoa1105243 21767103 PMC3200068

[13] Bazzi AR, Drainoni M, Biancarelli DL, et al. Systematic review of HIV treatment adherence research among people who inject drugs in the United States and Canada: Evidence to inform pre-exposure prophylaxis (PrEP) adherence interventions. BMC Public Health. 2019;19(1):31. 10.1186/s12889-018-6314-8 30621657 PMC6323713

[14] Rose RJ, Winter T, Viken RJ, Kaprio J. Adolescent alcohol use and adverse adult outcomes: Evaluating confounds with drinking-discordant twins. Alcohol Clin Exp Res. 2014;38(8):2314–2321. 10.1111/acer.12491 25040879 PMC4146743

[15] Waldron JS, Malone SM, McGue M, Iacono WG. Genetic and environmental sources of covariation between early drinking and adult functioning. Psychol Addict Behav. 2017;31(5):589–600. 10.1037/adb0000283 28594187 PMC5547004

[16] Paquette R, Tanton C, Burns F, et al. Illicit drug use and its association with key sexual risk behaviours and outcomes: Findings from Britain’s third National Survey of Sexual Attitudes and Lifestyles (Natsal-3). PLoS One. 2017;12(5):e0177922. 10.1371/journal.pone.0177922 28542366 PMC5436851

[17] Zibbell JE, Asher AK, Patel RC, et al. Increases in acute hepatitis C virus infection related to a growing opioid epidemic and associated injection drug use, United States, 2004–2014. Am J Public Health. 2018;108(2):175–181. 10.2105/AJPH.2017.304132 29267061 PMC5846578

[18] Davis A, McCrimmon T, Dasgupta A, et al. Individual, social, and structural factors affecting antiretroviral therapy adherence among HIV-positive people who inject drugs in Kazakhstan. Int J Drug Policy. 2018;62:43–50. 10.1016/j.drugpo.2018.08.014 30359872 PMC6279490

[19] Global Drug Policy Observatory. Country snapshot: Drugs in Zimbabwe [homepage on the Internet]. 2014 [cited 2022 Jun 15]. Available from: https://gdpo.swan.ac.uk/?p=245

